# Addendum: High economic costs of reduced carbon sinks and declining biome stability in Central American forests

**DOI:** 10.1038/s41467-025-57268-w

**Published:** 2025-02-28

**Authors:** Lukas Baumbach, Thomas Hickler, Rasoul Yousefpour, Marc Hanewinkel

**Affiliations:** 1 Chair of Forestry Economics and Forest Planning, University of Freiburg, Tennenbacherstr. 4, 79106 Freiburg, Germany; 2 Senckenberg Biodiversity and Climate Research Centre (BiK-F), Senckenberganlage 25, 60325 Frankfurt am Main, Germany; 3 Department of Physical Geography, Goethe University Frankfurt, Altenhöferallee 1, 60438 Frankfurt am Main, Germany; 4 Institute of Forestry and Conservation, John H. Daniels Faculty of Architecture, Landscape, and Design, University of Toronto, 33 Willcocks St, Toronto, ON M5S 3B3 Canada

Addendum to: *Nature Communications* 10.1038/s41467-023-37796-z, published online 11 April 2023

The application of discounting in environmental economics with the goal to estimate climate change induced losses is still subject to intense scientific debate (e.g., Nordhaus-Weitzmann-Stern debate; Nordhaus 2007 (ref. ^1^), Weitzman 2007 (ref. ^2^)). Central points to the discussion are ethical assumptions on intergenerational equity as well as uncertain projections of global economic growth and emission reduction costs. Omitting the discount rate altogether represents a scenario that accounts for the well-being of future generations, in which environmental damages are viewed as causing long-term losses. Since this behaviour is rarely observed in reality, classical economics uses discounting to take into account human preferences for short-term over long-term effects. Particularly in a global context, this discordance between environmental ethics and economic principles hampers the choice of an appropriate discount rate. Therefore, these limitations need to be kept in mind for interpreting economic estimates both with and without discounting.

Additionally, in the context of climate impacts on ecosystems and the services they provide, it needs to be noted that the underlying ecosystem processes are highly dynamic. Unlike in standard economic assessments, ecosystems do not provide continuous value flows and may behave in a nonlinear way (particularly habitat provision). To avoid such intricacies, in this study we look only at long-term trends and compare average conditions of two periods in time. This simplified approach, however, neglects additional value flows in between these periods. Therefore, the development of a more dynamic discounting method that accounts for nonlinear effects would be more desirable yet was beyond the scope of our study.
