# Supplementary material for: High economic costs of reduced carbon sinks and declining biome stability in Central American forests
Source: Nat Commun. 2023 Apr 11;14:2043. doi: 10.1038/s41467-023-37796-z (PMC10090148; doi:10.1038/s41467-023-37796-z)
Supplement: Supplementary file 1 — Supplementary Information [file 41467_2023_37796_MOESM1_ESM.pdf]

Supplementary

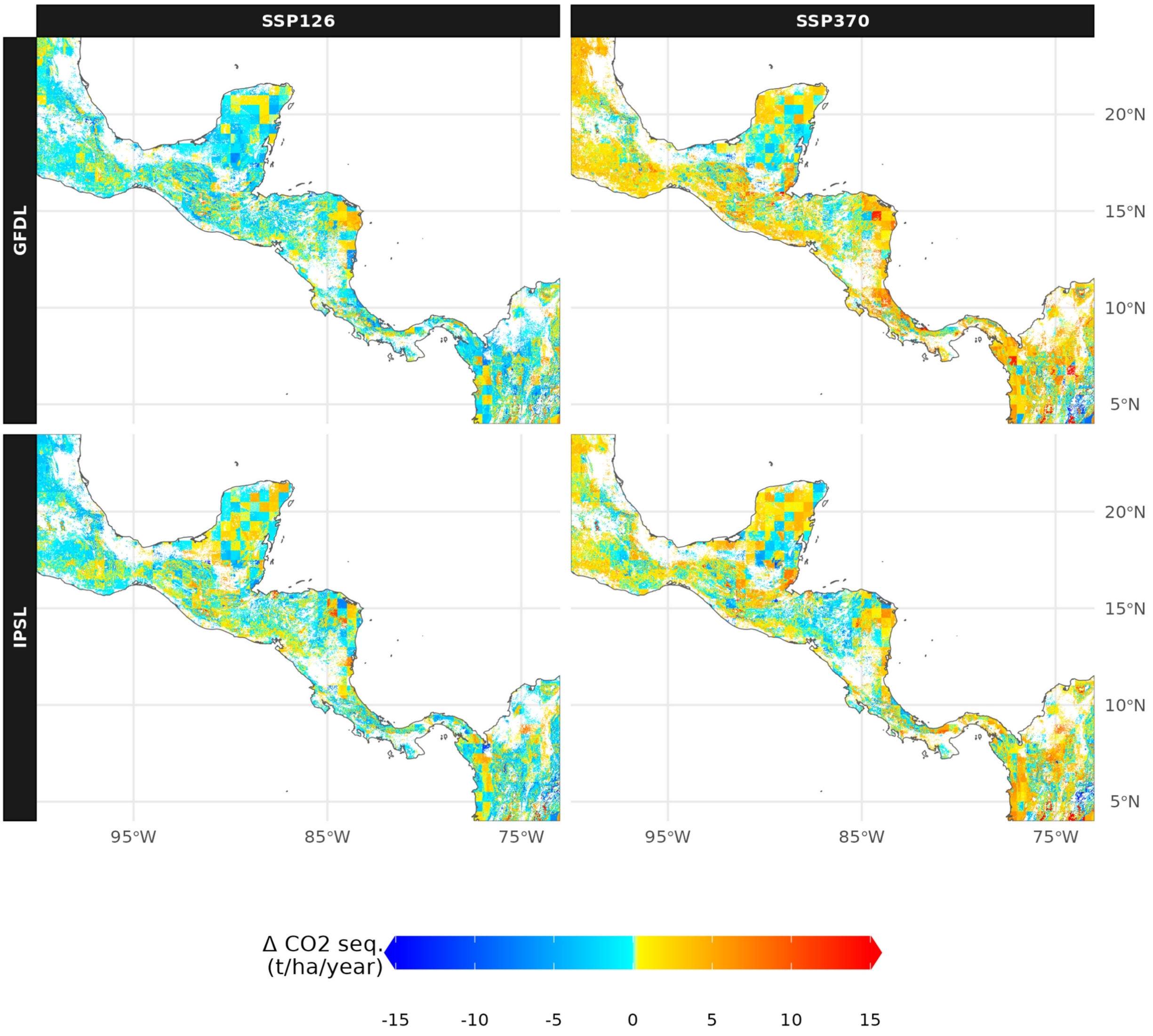

Figure S1: CO_2 sequestration difference between the reference (1985-2014) and future period (2071-2100).

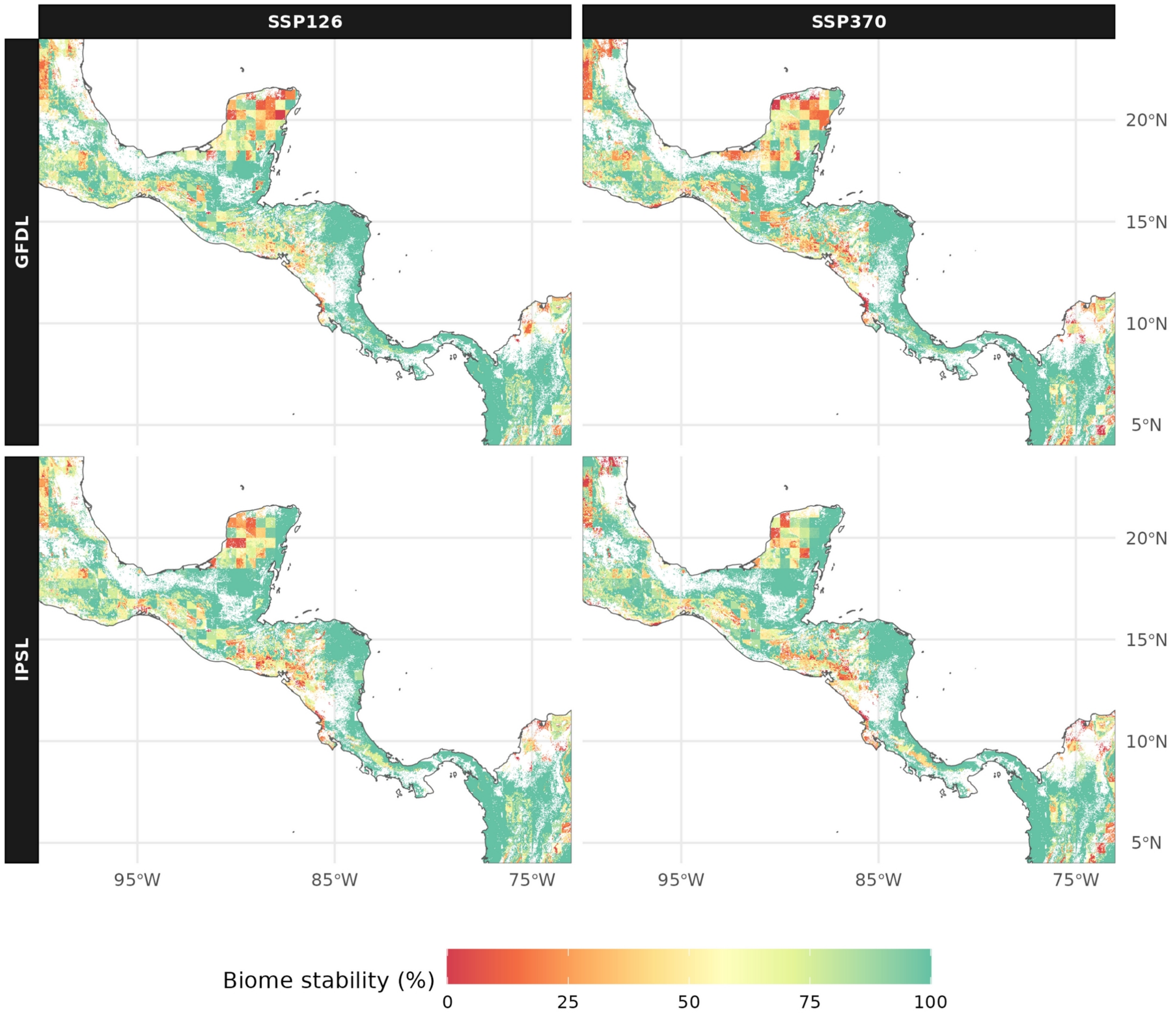

Figure S2: Biome stability projections for the future period (2071-2100).

Table S1: Area statistics (% of total area) for the bivariate map classes (B= biome stability, C = CO_2 sequestration)

	SSP126		SSP370	
	GFDL	IPSL	GFDL	IPSL
B<50% – C<0	8.72	8.17	4.02	4.93
B<50% – C>0	6.48	6.95	16.62	10.61
B>50% – C<0	55.33	47.74	20.06	28.43
B>50% – C>0	29.47	37.14	59.3	56.02

Table S2: Summary of economic valuation (HS = habitat services, CR= climate regulation, G= global price, N= national price)

**Scenario**	**discounting**	**HS loss area** (study area %)	**Cost of HS loss** (B$/year)		**CR loss area** (study area %)	**Cost of CR loss** (B$/year)		**Total cost** (B$/year)	
			G	N		G	N	G	N
**GFDL-SSP126**	none	54	49	31	64	65	39	114	70
	2%	100	310	186	36	2	1	313	187
**IPSL-SSP126**	none	54	48	29	56	56	33	104	62
	2%	100	310	185	24	2	1	312	186
**GFDL-SSP370**	none	56	58	36	24	31	17	89	53
	2%	100	313	187	9	2	1	314	188
**IPSL-SSP370**	none	52	48	28	33	43	23	91	51
	2%	100	310	185	15	3	1	313	186

Table S3: Ecosystem service losses in relation to national GDPs (year 2020), global prices (HS = habitat services, CR= climate regulation)

Country	Scenario	Discoun-ting	HS loss		CR loss		Total	
			M$/year	*%GDP*	*M$/year*	*%GDP*	*M$/year*	*%GDP*
Belize	GFDL-SSP126	none	-309	17.5	-1974	111.9	-2283	129.4
		2%	-5903	334.7	-57	3.3	-5961	337.9
	IPSL-SSP126	none	-138	7.8	-1346	76.3	-1484	84.1
		2%	-5861	332.2	-58	3.3	-5918	335.5
	GFDL-SSP370	none	-230	13.1	-1146	65	-1376	78
		2%	-5884	333.5	-18	1	-5902	334.6
	IPSL-SSP370	none	-281	15.9	-1221	69.2	-1502	85.2
		2%	-5897	334.3	-108	6.1	-6004	340.4
Colombia	GFDL-SSP126	none	-6839	2.5	-17074	6.3	-23913	8.8
		2%	-69943	25.8	-617	0.2	-70560	26
	IPSL-SSP126	none	-7473	2.8	-16288	6	-23761	8.8
		2%	-70101	25.8	-580	0.2	-70681	26
	GFDL-SSP370	none	-9358	3.4	-11238	4.1	-20596	7.6
		2%	-70572	26	-838	0.3	-71410	26.3
	IPSL-SSP370	none	-9228	3.4	-10572	3.9	-19800	7.3
		2%	-70540	26	-615	0.2	-71155	26.2
Costa Rica	GFDL-SSP126	none	-807	1.3	-2200	3.6	-3007	4.9
		2%	-11020	17.9	-105	0.2	-11125	18.1
	IPSL-SSP126	none	-1303	2.1	-2284	3.7	-3587	5.8
		2%	-11144	18.1	-87	0.1	-11232	18.3
	GFDL-SSP370	none	-1124	1.8	-644	1	-1768	2.9
		2%	-11100	18	-39	0.1	-11138	18.1
	IPSL-SSP370	none	-1718	2.8	-1987	3.2	-3705	6
		2%	-11248	18.3	-81	0.1	-11329	18.4
El Salvador	GFDL-SSP126	none	-1660	6.7	-540	2.2	-2200	8.9
		2%	-5101	20.7	-15	0.1	-5116	20.8
	IPSL-SSP126	none	-2057	8.3	-331	1.3	-2388	9.7
		2%	-5200	21.1	-5	0	-5205	21.1
	GFDL-SSP370	none	-1975	8	-21	0.1	-1995	8.1
		2%	-5180	21	-1	0	-5181	21
	IPSL-SSP370	none	-2010	8.2	-278	1.1	-2287	9.3
		2%	-5188	21.1	-5	0	-5194	21.1
Guatemala	GFDL-SSP126	none	-2654	3.4	-6116	7.9	-8770	11.3
		2%	-24074	31	-177	0.2	-24251	31.2
	IPSL-SSP126	none	-2884	3.7	-3635	4.7	-6519	8.4
		2%	-24132	31.1	-107	0.1	-24239	31.2
	GFDL-SSP370	none	-3796	4.9	-3123	4	-6919	8.9
		2%	-24360	31.4	-136	0.2	-24496	31.6
	IPSL-SSP370	none	-2735	3.5	-5018	6.5	-7753	10
		2%	-24094	31	-450	0.6	-24545	31.6
Honduras	GFDL-SSP126	none	-3379	14.2	-5729	24	-9107	38.2
		2%	-26961	113.1	-263	1.1	-27224	114.3
	IPSL-	none	-4712	19.8	-5188	21.8	-9899	41.5

	SSP126	2%	-27295	114.5	-174	0.7	-27468	115.3
	GFDL-SSP370	none	-4202	17.6	-3039	12.8	-7241	30.4
		2%	-27167	114	-219	0.9	-27386	114.9
	IPSL-SSP370	none	-4646	19.5	-7599	31.9	-12245	51.4
		2%	-27278	114.5	-621	2.6	-27899	117.1
Mexico	GFDL-SSP126	none	-30927	2.9	-23703	2.2	-54630	5.1
		2%	-129430	12	-893	0.1	-130323	12.1
	IPSL-SSP126	none	-25699	2.4	-19989	1.9	-45689	4.2
		2%	-128123	11.9	-633	0.1	-128756	12
	GFDL-SSP370	none	-34274	3.2	-9060	0.8	-43334	4
		2%	-130267	12.1	-432	0	-130699	12.1
	IPSL-SSP370	none	-22981	2.1	-11987	1.1	-34967	3.2
		2%	-127443	11.8	-731	0.1	-128174	11.9
Nicaragua	GFDL-SSP126	none	-2459	19.5	-3506	27.8	-5964	47.3
		2%	-23651	187.4	-153	1.2	-23804	188.6
	IPSL-SSP126	none	-3254	25.8	-3098	24.5	-6352	50.3
		2%	-23850	189	-83	0.7	-23932	189.6
	GFDL-SSP370	none	-3060	24.2	-1380	10.9	-4441	35.2
		2%	-23801	188.6	-75	0.6	-23876	189.2
	IPSL-SSP370	none	-3337	26.4	-3265	25.9	-6602	52.3
		2%	-23871	189.1	-292	2.3	-24162	191.4
Panama	GFDL-SSP126	none	-236	0.4	-3764	7.1	-4000	7.6
		2%	-14282	27	-167	0.3	-14450	27.3
	IPSL-SSP126	none	-465	0.9	-3420	6.5	-3886	7.3
		2%	-14340	27.1	-85	0.2	-14425	27.2
	GFDL-SSP370	none	-248	0.5	-1057	2	-1304	2.5
		2%	-14285	27	-45	0.1	-14330	27.1
	IPSL-SSP370	none	-655	1.2	-1467	2.8	-2122	4
		2%	-14387	27.2	-72	0.1	-14459	27.3
All	GFDL-SSP126	none	-310366	19.4	-2447	0.2	-312813	19.5
		2%	-49270	3.1	-64605	4	-113874	7.1
	IPSL-SSP126	none	-310045	19.3	-1811	0.1	-311857	19.5
		2%	-47986	3	-55578	3.5	-103564	6.5
	GFDL-SSP370	none	-312616	19.5	-1801	0.1	-314417	19.6
		2%	-58267	3.6	-30706	1.9	-88973	5.6
	IPSL-SSP370	none	-309946	19.3	-2975	0.2	-312921	19.5
		2%	-47590	3	-43393	2.7	-90983	5.7

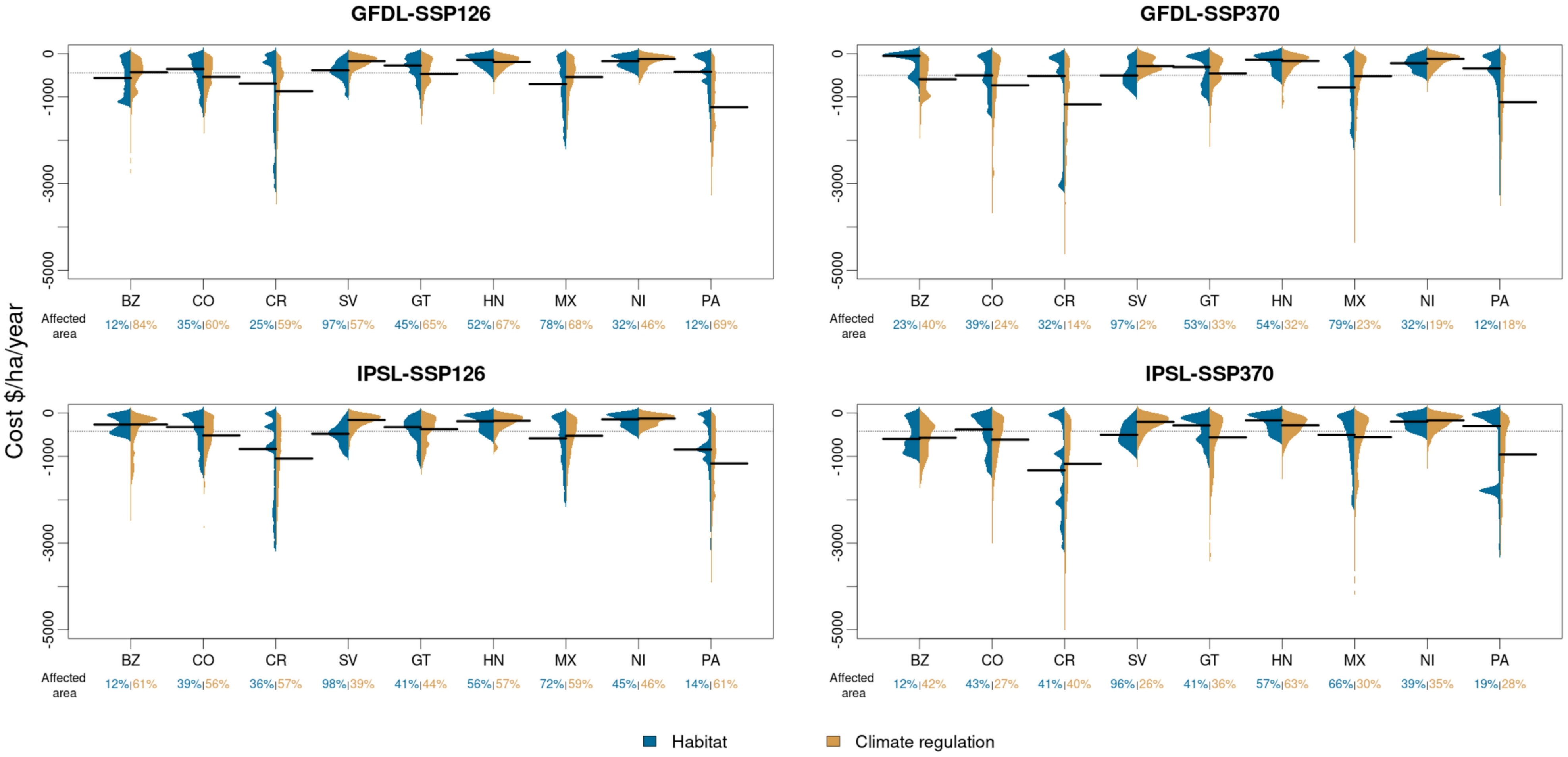

Figure S3: Country-level distribution of ecosystem service losses: national prices, no discounting. Bean plots showing the economic costs for declining habitat services (blue color) and decreased climate regulation (dark yellow) separated by country (BZ=Belize, CO=Colombia, CR=Costa Rica, SV=El Salvador, GT=Guatemala,HN=Honduras, MX=Mexico, NI=Nicaragua, PA=Panama). The affected area shares for each country are relative to the respective national total forest area within the study extent. The dotted line shows the overall average for each scenario.

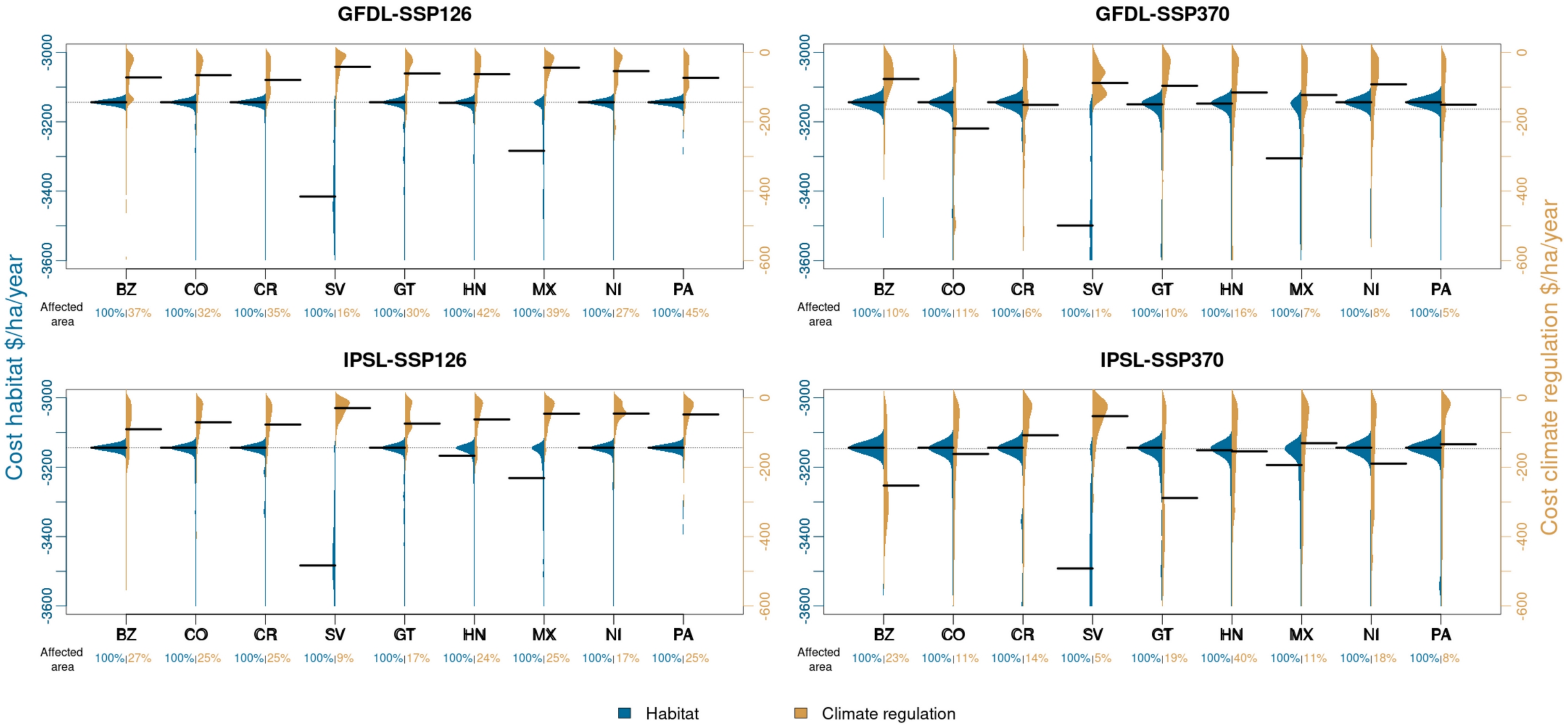

Figure S4: Country-level distribution of ecosystem service losses: global prices, 2% discount rate. For details see Fig. S3.

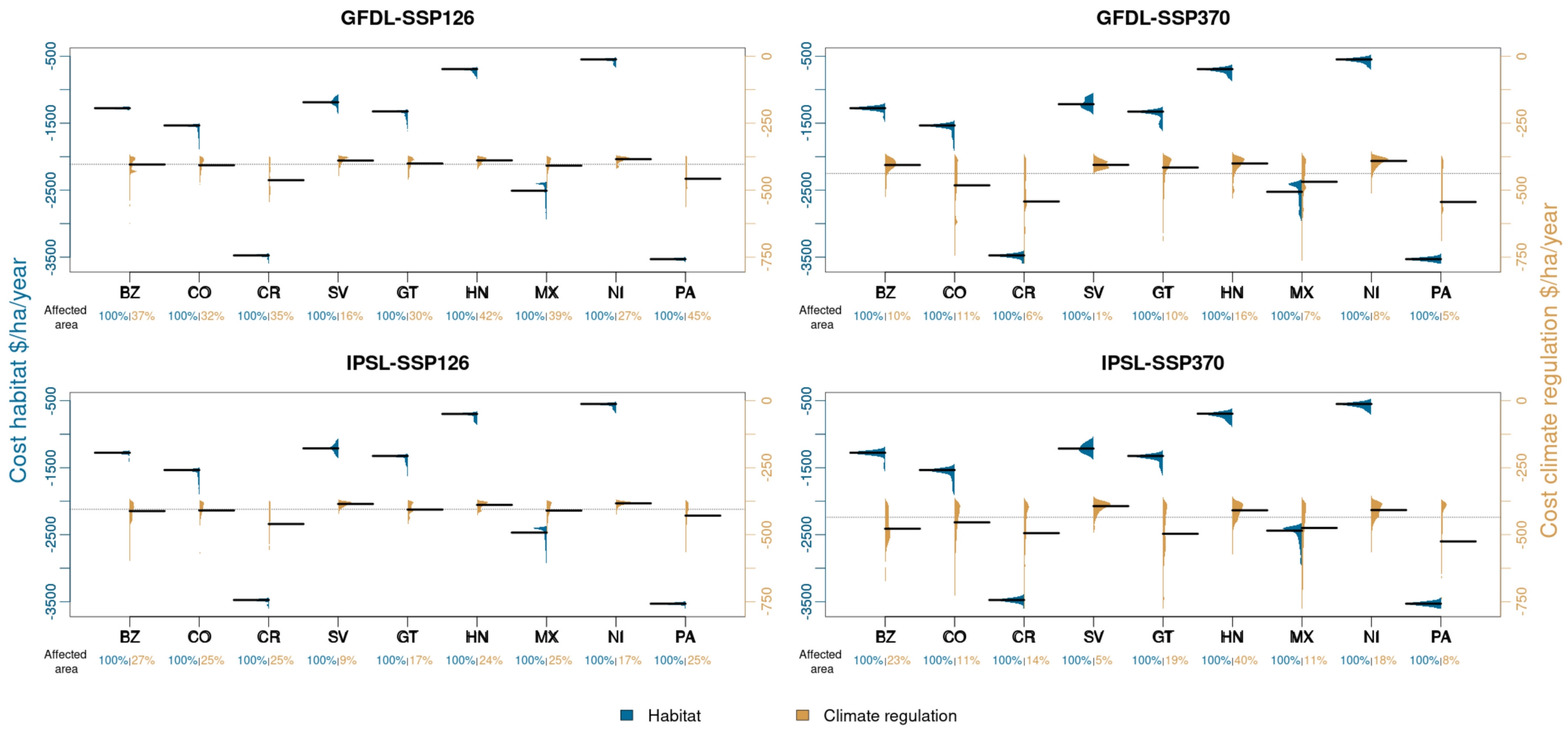

Figure S5: Country-level distribution of ecosystem service losses: national prices, 2% discount rate. For details see Fig. S3.

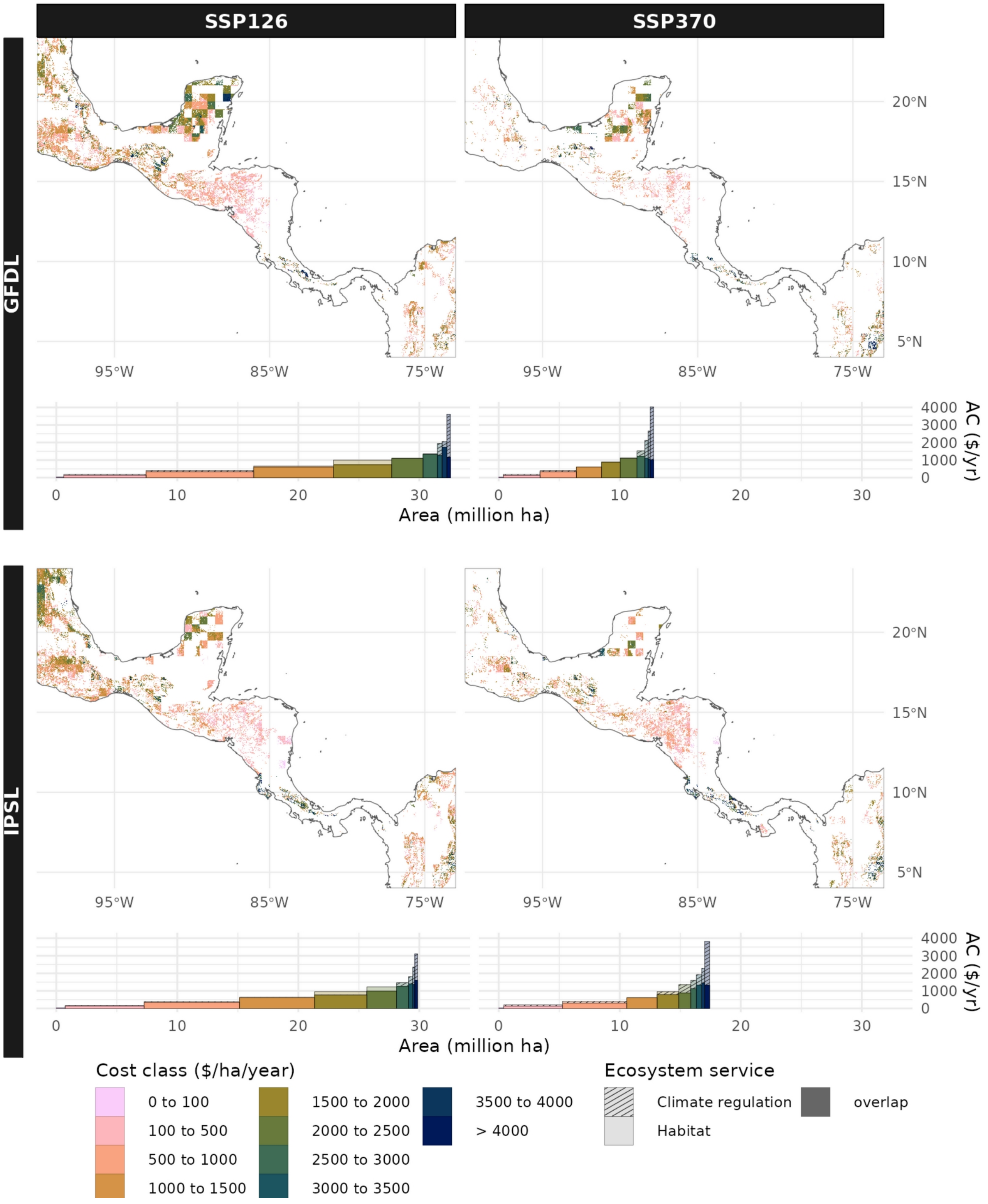

Figure S6: Economic hot spots: national prices, no discounting. The here presented maps show projected economic losses in areas where both ES declined. Cost classes refer to the summed costs of declines in both services. The bar plots show an overlay of the individual ES costs to compare their contribution to overall costs. Bar width shows the area covered by that class, bar height the average cost (AC).

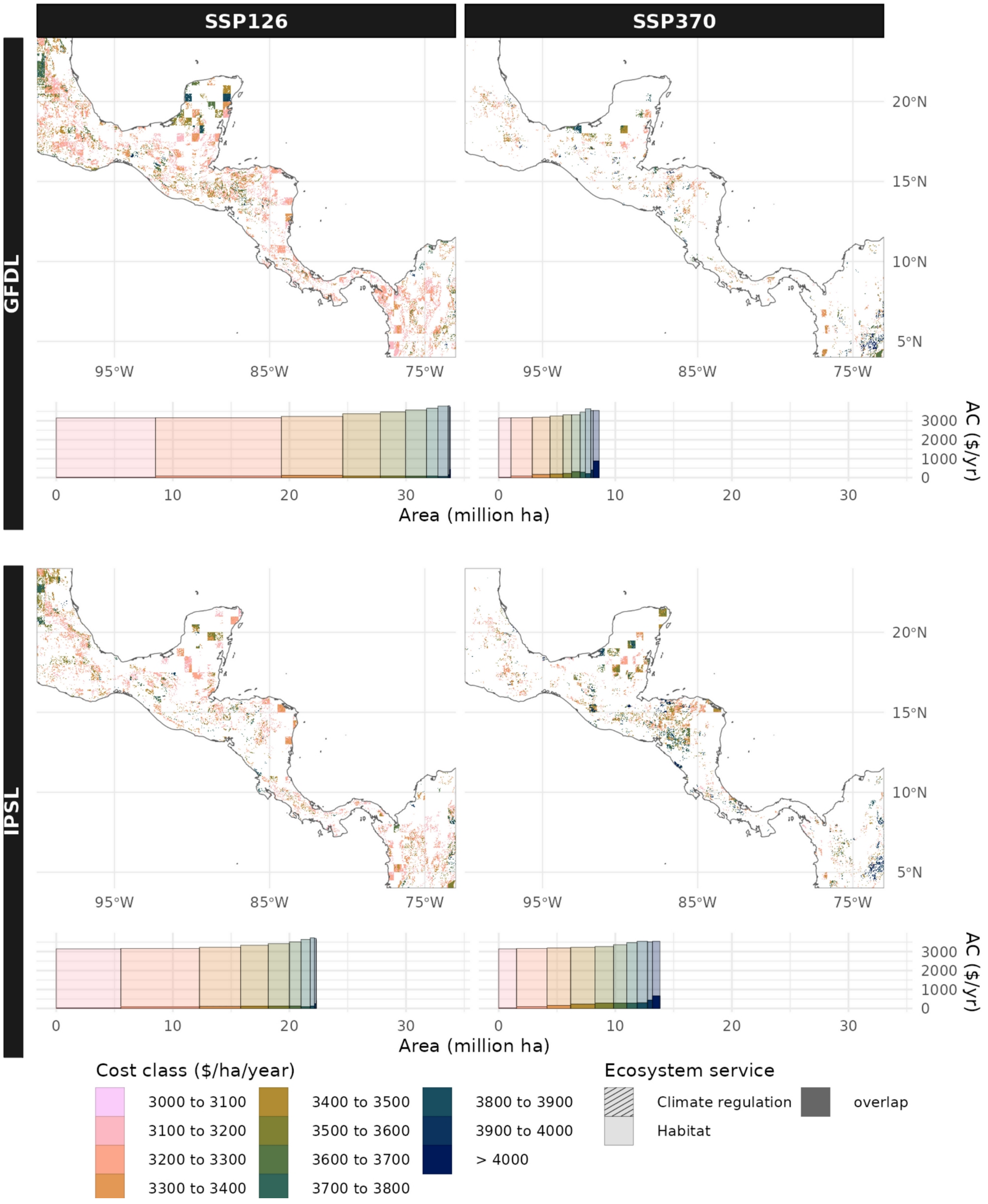

Figure S7: Economic hot spots: global prices, 2% discount rate. For details see Fig. S6.

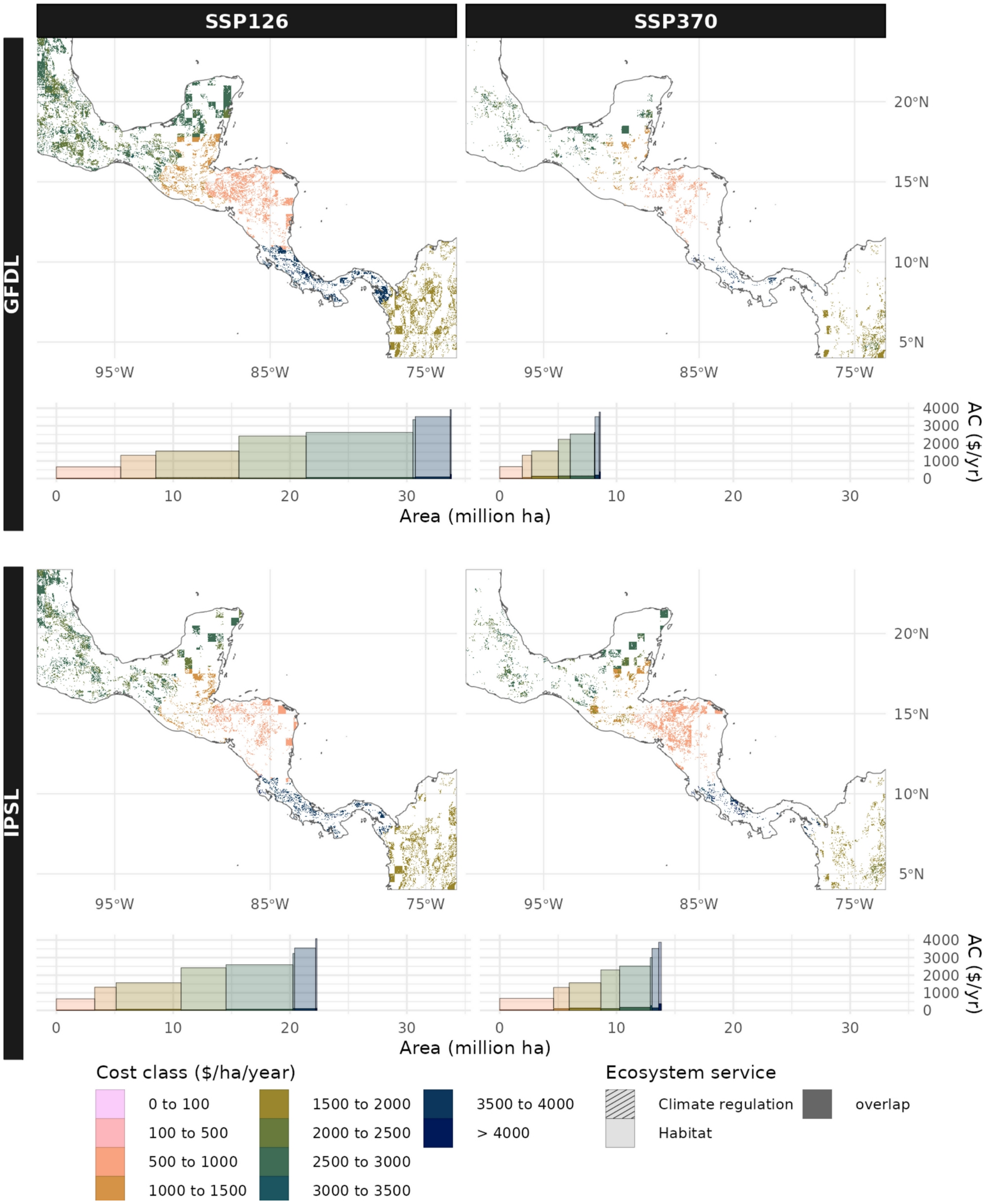

Figure S8: Economic hot spots: national prices, 2% discount rate. For details see Fig. S6.

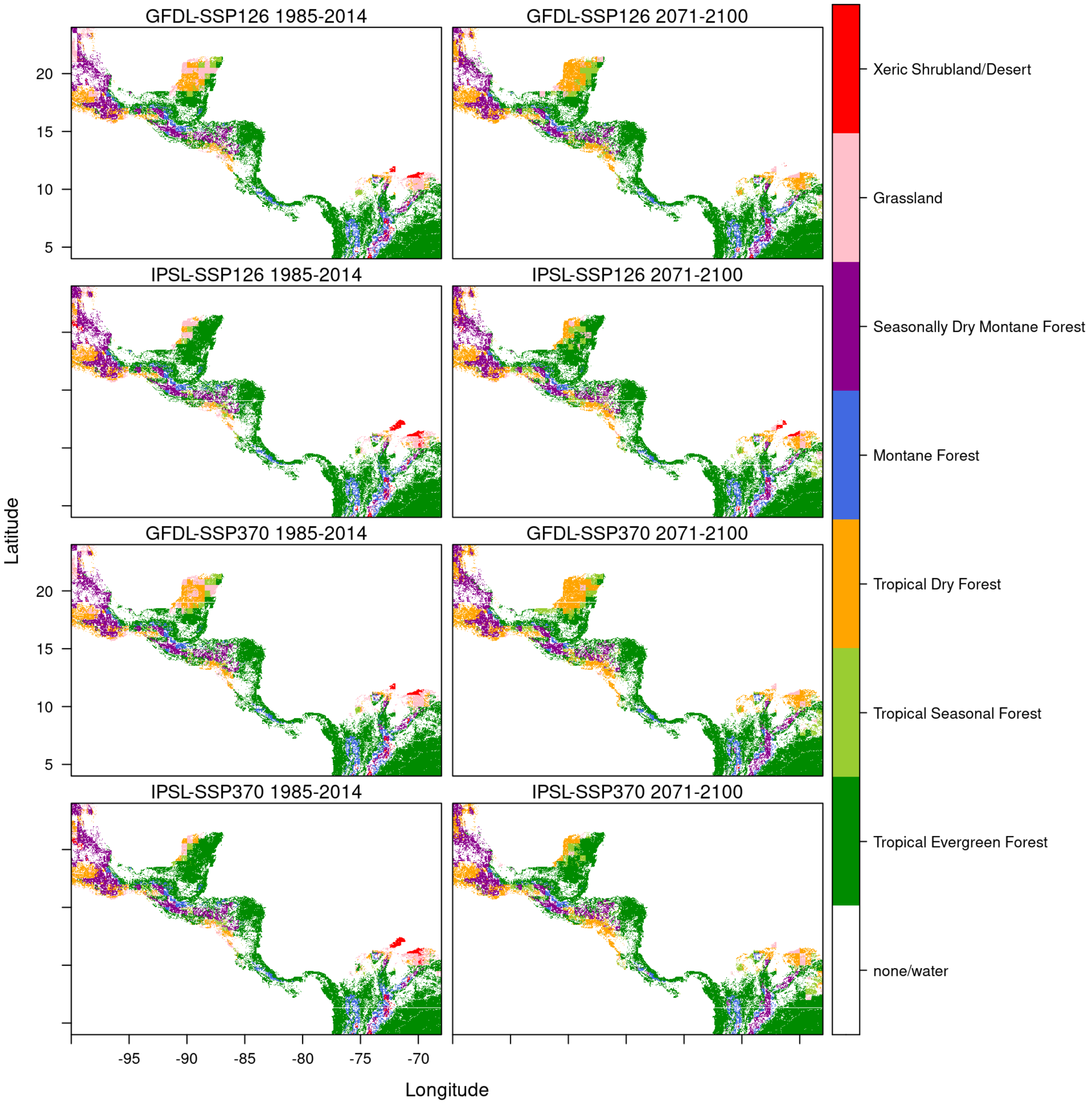

Figure S9: Average biomes for the historical (1985-2014) and future period (2071-2100).

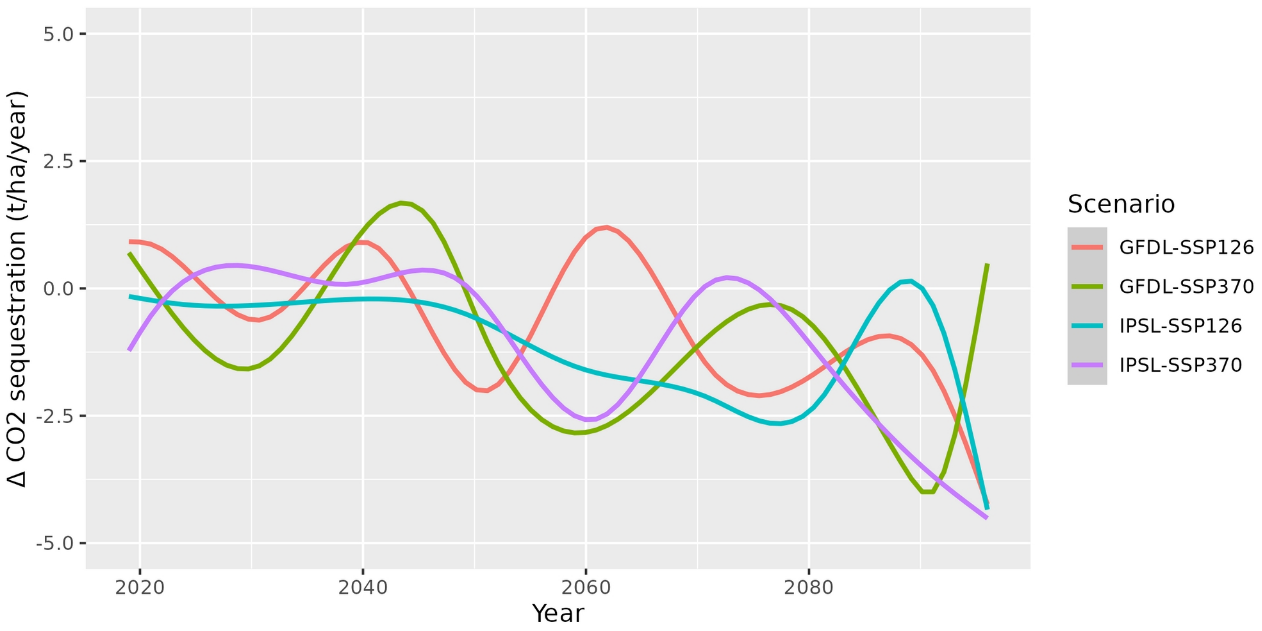

Figure S10: Temporal development of CO_2 sequestration in areas where both ecological indicators declined. Lines show the smoothed mean for each scenario.

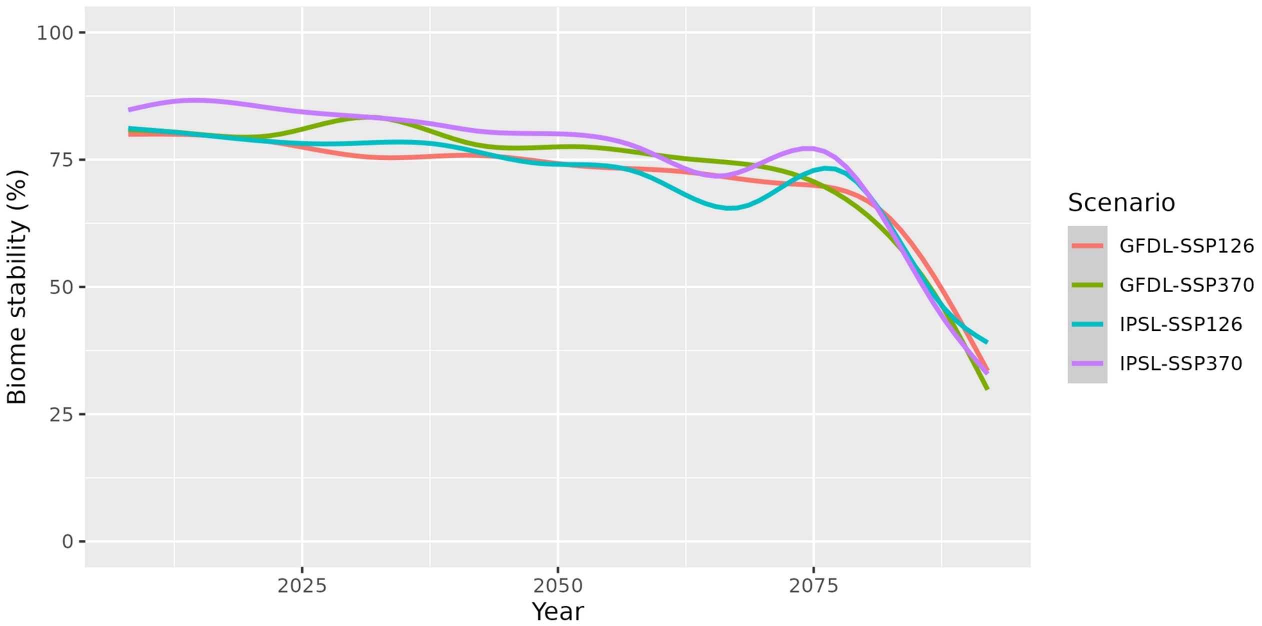

Figure S11: Temporal development of biome stability in areas where both ecological indicators declined. Lines show the smoothed mean for each scenario.

Supplementary methods

Detailed model description

LPJ-GUESS supports different vegetation modes from individual plants to whole populations to represent processes at varying scales. Here we used the cohort mode, which is aimed at representing forest stands by simulating individuals in replicate patches of 0.1ha each, "corresponding in size approximately to the maximum area of influence of one large adult individual (usually a tree) on its neighbours" [1]. The standard version of LPJ-GUESS includes global PFT parametrizations, of which five may be found within Central America: (1) Tropical broadleaved evergreen forest (TrBE and its shade-intolerant counterpart TrIBE), which is the presently dominant biome throughout large parts of the study region; (2) tropical broadleaved raingreen forest (TrBR), which refers to plant types adapted to seasonal drought (Central American dry forest ecoregions); (3) temperate broadleaved evergreen forests (TeBE), which may be found in high elevations with temporal freezing conditions; (4) C3 grasses (C3), which cover parts of the higher mountain ranges as parámo grasslands; and (5) C4 grasses, which are adapted to high tempratures and thrive in savanna-like landscapes (e.g. Mexico, parts of the Pacific coast). PFT-specific settings are summarized in Table S3.1, general settings in Table S3.2.

To account for the effect of topography within the coarse 0.5° resolution climate inputs, we adapted the landforms approach first introduced by Werner et al. [2]. The main idea of this method is to adjust climate inputs based on fundamental geophysical relationships of topography with temperature and insolation. As temperature decreases with elevation and insolation depends on aspect, slope and time of the year, these causalities can be used to adapt climate inputs at the fine resolution of digital elevation models (DEMs). While a high spatial resolution allows for a precise representation of gradients in mountain ranges, the computational effort for modelling is high and potentially not well-invested for large homogenous landscapes. Therefore, each grid cell is classified into so-called "landforms" based on elevation bands of 200m and the topographic position index, which discriminates between ridges, mid-slope positions and valleys [3]. To further reduce the computational effort, rare landforms covering less than 1% of each gridcell were excluded from the analysis. The final classification yielded between 2 and 189 (mean: 51.2) landform classes for each grid cell. Each landform was run as a subversion of the original grid cell (implemented as stand) with adjusted climate inputs based on the landform properties. Therefore, we calculated a regionalized mean lapse rate of -5.2K/km based on elevation and downscaled temperature data (CHELSA v1.2) [4,5] and adjusted temperatures for each landform accordingly. Solar radiation was adjusted based on

solar angle, slope and aspect [2]. Finally, we modified each landform's soil depth based on the topographic position index (ridges: 0.75 m, mid-slope: 1 m, and valleys 2 m).

With these adjusted settings we ran our simulations for the above described PFTs for each stand (landform) with 15 replicate patches each.

Model evaluation and adaptation

For a basic evaluation of model performance we first tested LPJ-GUESS with default settings and compared the outputs to satellite-derived data and maps. Biome types were derived following the biome classification scheme of Snell et al. [6] and compared to the biome map by Olson et al. [7]. In terms of biome distribution, the default settings with activated fire module (GlobFIRM) [8] led to an overrepresentation of grasses and a low productivity of the tropical rain green PFT. A scatterplot of simulated fire season length vs. fire observations (GFED 4) [9] showed a bell-shaped distribution of the data points, i.e. lowest fire probability both at very short and very long simulated fire season length. If the fire module was turned off on the other hand, biome distribution largely agreed with the benchmark map. Since a large share of fires are actively managed in Central America [10] and overall burnt area was small compared to the total area of the study region, we proceeded without fire modeling (also compare Snell et al. [6]).

A comparison between simulated and satellite-derived NPP (MODIS-NPP) [11] further revealed an underestimation, particularly in montane regions. In relation to this, Atkin et al. [12] pointed out, that the thermal acclimation of respiration can play a significant role for plant growth, yet is rarely considered in dynamic vegetation models. To better account for this fact, we adapted the respiration function in our model code based on adjustments by Dantas de Paula et al. [13] and Thum et al. [14]. Finally, we compared carbon mass outputs to estimations of above- and belowground carbon in vegetation by Spawn and Gibbs [15]. For most areas, our model simulations strongly exceeded the satellite-based predictions (RMSE > 16). Yet, the largest discrepancies also fell together with the areas with the highest uncertainty in the compared map. In addition to this, the standard LPJ-GUESS disturbance interval of 200 years allowed for a long accumulation period of carbon. Opposed to this, the region is quite regularly affected by disturbances like tropical storms, floods, land slides and droughts [16] – also in combination with El Niño/La Niña dynamics – and may be more so in the future [17]. Therefore, we decided to reduce the disturbance interval to 100 years. With these adjusted settings, our model showed moderate to good agreement with satellite-derived products and biome maps and was able to reproduce major spatial patterns (see Figures S8-9).

Table S4: List of datasets used as model drivers and for model evaluation

	Variable(s)	Short name	Data set, source	Data type	Spatial resolution	Temporal resolution and coverage
Model drivers	Near-surface air temperature	tas	ISIMIP 3b, Lange (2020, 2019)	Model output	0.5°	daily 1850-2100
	Precipitation	pr	ISIMIP 3b, Lange (2020, 2019)	Model output	0.5°	daily 1850-2100
	Surface downwelling shortwave radiation	rsds	ISIMIP 3b, Lange (2020, 2019)	Model output	0.5°	daily 1850-2100
	Atmospheric CO2 concentration	co2	Meinshausen et al. (2020)	Model output	northern hemisphere	Yearly 1-2500
	Nitrogen deposition	ndep	ACCMIP, Lamarque et al. (2013)	Model output	0.5°	decadal 1850-2100
	Elevation, slope, aspect	-	SRTMGL1, NASA JPL (2013)	Satellite data	1 arc-second	2000
Model evaluation	Net primary production	NPP	MODIS-NPP	Satellite-derived	500 m	2003-2019
	Carbon mass	-	Spawn et al. (2020)	Satellite-derived	300 m	2010
	Biome type	-	Olson et al. (2001)	map	30 arc-second	-
	Land cover		ESA (2020)	Satellite-derived	300 m	2019

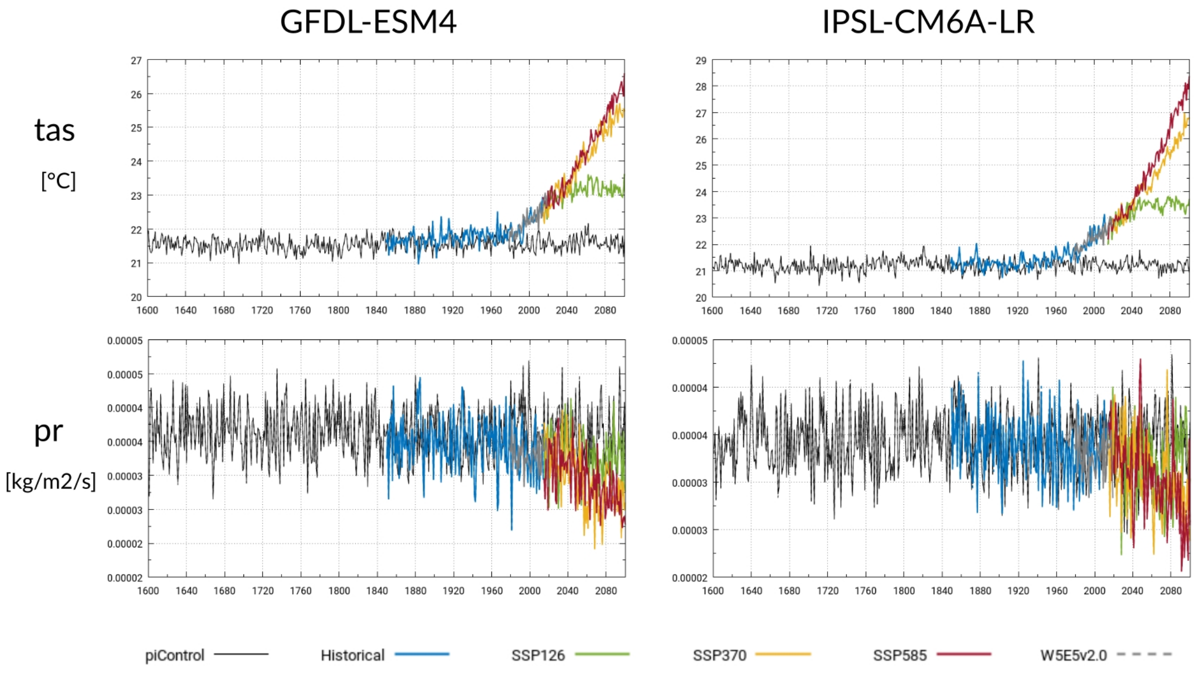

Fig. S12: Average yearly climate input data (tas = temperature average surface, pr = precipitation) for the Central American region under different scenarios (piControl = pre-industrial emission levels, W5E5v2.0 = observational dataset for comparison). Edited from ISIMIP Data Team 2022.

Table S5.1: LPJ-GUESS PFT parameter settings (blue=PFT-specific, red=lifeform-specific, orange=climate zone traits, green= leaf traits, yellow= shade tolerance traits)

	**parameter**	**TrBE**	**TrIBE**	**TrBR**	**TeBE**	**C3G**	**C4G**
Lifeform	**lifeform**	Tree	Tree	Tree	Tree	Grass	Tree
	**rootdist(upper)**	0.6	0.6	0.6	0.6	0.9	0.9
	**rootdist(lower)**	0.4	0.4	0.4	0.4	0.1	0.1
	**km_volume ***	$14.77*10^{-7}$	$14.77*10^{-7}$	$14.77*10^{-7}$	$14.77*10^{-7}$	$18.76*10^{-7}$	$18.76*10^{-7}$
	**Nuptoroot ***	0.0028	0.0028	0.0028	0.0028	0.00551	0.00551
climate	**climate**	tropical	tropical	tropical	temperate		
	**pstemp_high**	30	30	30	25	30	45
	**pstemp_low**	25	25	25	15	10	20
	**pstemp_max**	55	55	55	38	45	55
	**pstemp_min**	2	2	2	-2	-5	6
	**respcoeff**	0.15	0.15	0.15	1.0	1.0	0.15
	**tcmax_est**	1000	1000	1000	18.8	1000	1000
	**tcmin_est**	15.5	15.5	15.5	0	-1000	15.5
	**tcmin_surv**	15.5	15.5	15.5	-1	-1000	15.5
	**twmin_est**	-1000	-1000	-1000	5	-1000	-1000
Leaf	**phenology**	evergreen	evergreen	raingreen	evergreen	any	any
	**Fnstorage ***	0.05	0.05	0.15	0.05	0	0.3
	**phengdd5ramp**	0	0	0	200		
Shade tolerance	**Shade tolerance**	tolerant	intolerant	intolerant	tolerant		
	**alphar**	3.0	10.0	10.0	3.0		
	**est_max**	0.05	0.2	0.2	0.05		
	**greff_min**	0.04	0.08	0.08	0.04		
	**parff_min**	350000	2500000	2500000	350000		
PFT specific	**eps_iso ***	24.0	24.0	45.0	24.0	16.0	8.0
	**eps_mon ***	0.8	0.8	2.4	1.6	1.6	2.4
	**gdd5min_est**				2000		0
	**leaflong**	2	2	0.5	3	0.5	0.5
	**longevity**	500	200	400	300		
	**pathway**	c3	c3	c3	c3	c3	c4
	**seas_iso ***	0	0	0	0	1	
	**storfrac_mon ***	0	0	0	0	0.5	0.5
	**turnover_leaf**	0.5	0.5	1	0.33	1	1
	**turnover_sap**	0.05	0.1	0.1	0.05		

Table S5.2: LPJ-GUESS lifeform parameter settings

parameter	Tree PFTs	Grasses	parameter	Tree PFTs	Grasses
**aphen_max**	210	210	**k_chillb**	100	-
**cton_root**	29	29	**k_chillk**	0.05	-
**cton_sap**	330	-	**k_latosa**	6000	-
**crownarea_max**	40	-	**k_rp**	1.6	-
**drought_tolerance**	0.0001	0.0001	**kest_bg**	0.1	-
**emax**	5	5	**kest_repr**	200	-
**ga**	0.04	0.03	**lambda_max**	0.8	0.8
**gmin**	0.5	0.5	**ltor_max**	1	0.5
**intc**	0.02	0.01	**Nrelocfrac ***	0.5	0.5
**k_allom1**	250	-	**turnover_root**	0.7	0.7
**k_allom2**	60	-	**reprfrac**	0.1	0.1
**k_allom3**	0.67	-	**wooddens**	200	-
**k_chilla**	0	-	**wscal_min**	0.35	0.35

* additional parameters introduced by Werner et al. (2018)

Table S6: Biomization scheme. Dry months are defined as months with <100 mm precipitation.

**Biome**	**Conditions**
Tropical Broadleaved Evergreen Forest	Total LAI > 2.5 *and* TrBE LAI ≥ 66% Total LAI
Tropical Seasonal Forest	Total LAI > 2.5 *and* TrBE LAI ≥ 33% Total LAI *and* TrBR LAI ≥ 33% Total LAI
Tropical Dry Forest	Total LAI > 2.5 *and* TrBR LAI ≥ 66% Total LAI
Tropical Montane Forest	Total LAI > 2.5 *and* TeBE LAI > 10% Total LAI *and* TeNE LAI < 25% Total LAI *and* Number of dry months < 5
Seasonally Dry Montane Forest (pine-oak)	Total LAI > 2.5 *and* TeBE LAI > 10% Total LAI *and* Number of dry months ≥ 5
Savanna	Total LAI > 1.5 *and* (Grass NPP / Tree NPP) < 1.8 *and* C4 LAI > C3 LAI
Grassland	Total LAI > 1.5 *and* (Grass NPP / Tree NPP) > 1.8 *and* C4 LAI > C3 LAI ***or*** Total LAI > 3.0 *and* C3 LAI ≥ 66% Total LAI
Xeric woodland	Tree LAI > 0.5 *and* Tree LAI < 2.5 *and* Grass LAI < Tree LAI
Desert/Arid shrubland	Total LAI < 0.5

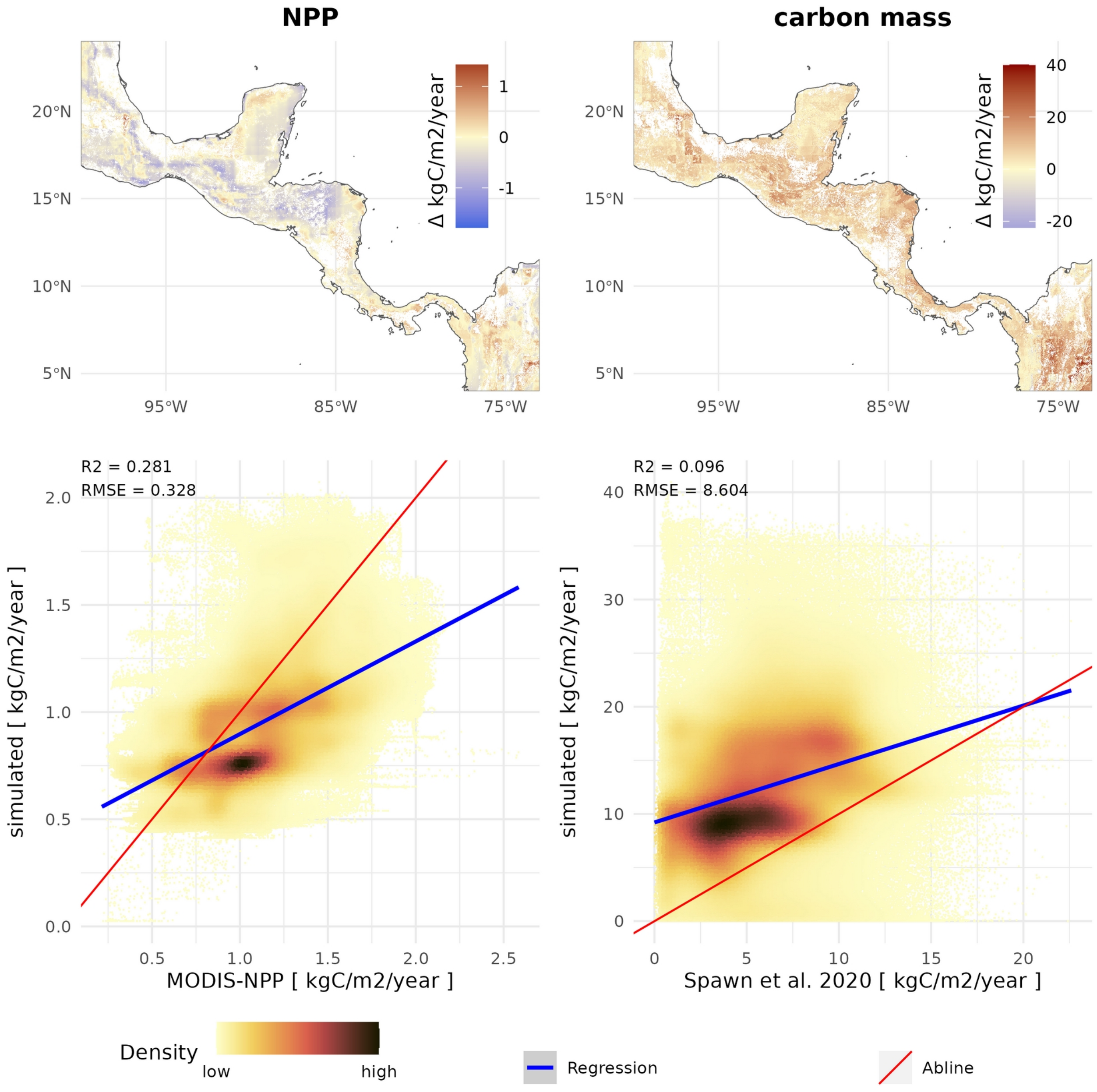

Figure S13: Comparison of LPJ-GUESS outputs (average over all runs) with satellite-derived products (MODIS-NPP and carbon mass estimates from Spawn et al. 2020).

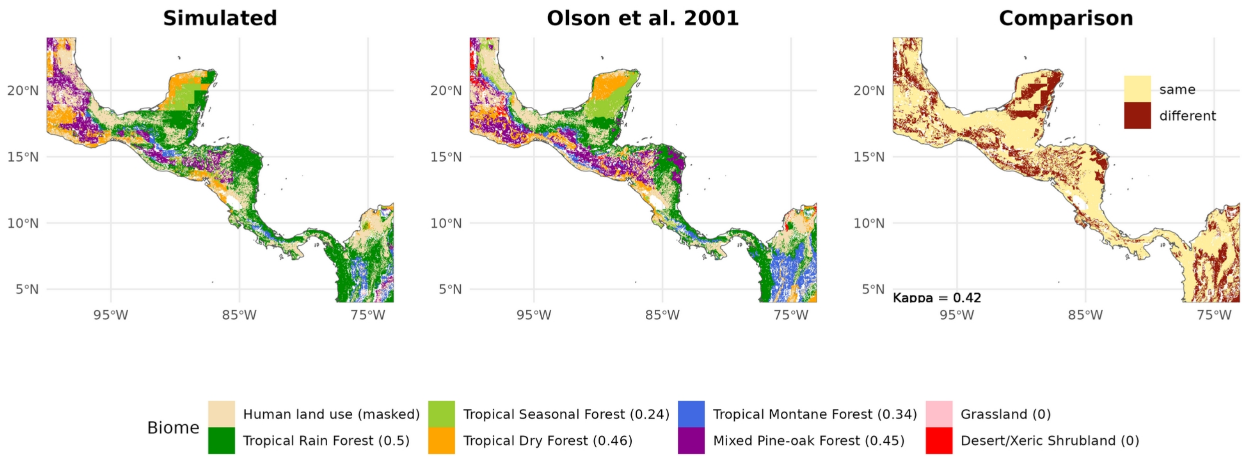

Figure S14: Comparison of LPJ-GUESS biomization (mean over the years 1985-2014) with biome classification by Olson et al. (2001). Overall kappa is shown in the comparison map, while individual kappa statistics of each biome are shown in brackets in the biome legend.

Ecosystem service valuation

The following filters were used to select data from the Ecosystem Service Valuation Database:

- **continents**: "South America", "Asia" and "Africa"
- **ecosystems**: "Tropical rain forest","Wetlands, Forested (on alluvial soils)", "Tropical cloud forests","Temperate rain or evergreen forest","High Mountain – forest", "Temperate deciduous forest", "Tropical dry forest","Savanna","Tropical grasslands","Pastures","High mountain - grassland", "Other (grassland)", "Steppe (dry, cold grassland)", "Temperate grasslands")
- **ecosystem services**: "Existence, bequest values","Opportunities for recreation and tourism", "Regulation of water flows","Maintenance of genetic diversity", "Moderation of extreme events", "Inspiration for culture, art and design" , "Erosion prevention", "Ornamental resources","Information for cognitive development","Maintenance of life cycles","Maintenance of soil fertility","Air quality regulation" ,"Waste treatment" "Pollination" ,"Biological control" ,"Genetic resources"

The resulting data selection comprised 246 records. The records were grouped by biomes to balance biases towards certain ecosystems (i.e. tropical rain forest). Overall characteristics of the final data set are summarized in Table S7.

Table S7: Characteristics of selected ESVD data

**Biome**	**Ecosystems (no. studies)**	**Ecosystem services**	**Valuation methods**[a]
Tropical rain forest	Tropical rain forest (178), Wetlands, Forested (on alluvial soils) (2)	Biological control, Erosion prevention, Existence/bequest values, Genetic resources, Information for coginitive development, Inspiration for culture, art and design, Maintenance of genetic diversity, Maintenance of life cycles, Maintenance of soil fertility, Moderation of extreme events, Opportunities for recreation and tourism, Ornamental resources, Pollination, Regulation of water flows, Waste treatment	CE, CV, DE, FI, MP, PF, DC, OC, RC, RT, TC, VT
Montane forest	Tropical cloud forests (2), Temperate rain or evergreen forest (12), High Mountain – forest (7)	Air quality regulation, Erosion prevention, Existence/bequest values, Information for cognitive development, Maintenance of soil fertility, Opportunities for recreation and tourism	CV, PP, DC, OC, RC, RT, TC, VT
Temperate forest	Temperate deciduous forest (26)	Air quality regulation, Erosion prevention, Maintenance of soil fertility, Opportunities for recreation and tourism, Regulation of water flows, Waste treatment	CV, HP, DC, OC, RC, RT, VT
Tropical dry woodlands	Tropical dry forest (1), Savanna (3)	Existence/bequest values, Maintenance of genetic diversity, Opportunities for recreation and tourism, Regulation of water flows	CE, CV, MP
Grassland	Tropical grasslands (2), Temperate grasslands (3), Steppe (dry, cold grassland) (5), Other (grassland) (5)	Air quality regulation, Erosion prevention, Existence/bequest values, Maintenance of genetic diversity, Maintenance of soil fertility	CV, FI, DC, OC, RT, VT

[a] Valuation methods: CE = Choice modelling, CV = Contingent valuation, DE = Defensive expenditure, FI = Net factor income, HP = Hedonic pricing, MP = Market prices, PP = Public pricing, PF = Production function, DC = Damage cost avoided, OC = Opportunity cost, RC = Replacement cost, RT = Restoration cost, TC = Travel cost, VT = Value transfer

Table S8: Overview of the applied ecosystem service values in dependence of socio-economic scenario, discount rate and time period. Ecosystem service value units are given in $ per t CO_2 for CO_2 sequestration and in $/ha/year for biome stability with standard deviations in brackets.

			**Ecosystem service value [$ per unit]**	
**indicator**	**scenario(s)**	**discounting**	**1985-2014**	**2071-2100**
CO_2 sequestration	SSP1	none	504 (±*332*)	504 (±*332*)
CO_2 sequestration	SSP1	2%	5 (±*14*)	62 (±*54*)
CO_2 sequestration	SSP3	none	650 (±*233*)	650 (±*233*)
CO_2 sequestration	SSP3	2%	20 (±*20*)	140 (±*91*)
Biome stability	all	none	2800 (±*1671*)	2800 (±*1671*)
Biome stability	all	2%	3844 (-)	700 (-)

Table S9: GDP 2020 [18], GDP per capita 2020 [19] and derived scaling factor for national CO_2 prices

Country	GDP year 2020 (million USD)	GDP per capita year 2020 (USD)	Scaling factor
Colombia	271347	5333	0.49
Panama	52938	12269	1.12
Costa Rica	61521	12077	1.11
Nicaragua	12621	1905	0.17
Honduras	23828	2406	0.22
El Salvador	24639	3799	0.35
Guatemala	77605	4603	0.42
Belize	1764	4436	0.45
Mexico	1076163	8347	0.76
World	84705426	10926	1

Supplementary references

1. Smith, B., Prentice, I. C. & Sykes, M. T. Representation of vegetation dynamics in the modelling of terrestrial ecosystems: comparing two contrasting approaches within European climate space. *Global Ecology and Biogeography* **10**, 621–637 (2001).
2. Werner, C. *et al.* Effect of changing vegetation and precipitation on denudation – Part 1: Predicted vegetation composition and cover over the last 21 thousand years along the Coastal Cordillera of Chile. *Earth Surface Dynamics* **6**, 829–858 (2018).
3. Weiss, A. Topographic position and landforms analysis. in vol. 200 (2001).
4. Karger, D. N. *et al.* Data from: Climatologies at high resolution for the earth's land surface areas, Dryad, Dataset. *Dryad* (2018) doi:https://doi.org/10.5061/dryad.kd1d4.
5. Karger, D. N. *et al.* Climatologies at high resolution for the earth's land surface areas. *Sci Data* **4**, 170122 (2017).
6. Snell, R. S., Cowling, S. A. & Smith, B. Simulating Regional Vegetation-climate Dynamics for Middle America: Tropical Versus Temperate Applications. *Biotropica* **45**, 567–577 (2013).
7. Olson, D. M. *et al.* Terrestrial Ecoregions of the World: A New Map of Life on EarthA new global map of terrestrial ecoregions provides an innovative tool for conserving biodiversity. *BioScience* **51**, 933–938 (2001).
8. Thonicke, K., Venevsky, S., Sitch, S. & Cramer, W. The role of fire disturbance for global vegetation dynamics: coupling fire into a Dynamic Global Vegetation Model. *Global Ecology and Biogeography* **10**, 661–677 (2001).
9. Giglio, L., Randerson, J. T. & van der Werf, G. R. Analysis of daily, monthly, and annual burned area using the fourth-generation global fire emissions database (GFED4). *Journal of Geophysical Research: Biogeosciences* **118**, 317–328 (2013).
10. Ríos, B. & Raga, G. B. Spatio-temporal distribution of burned areas by ecoregions in Mexico and Central America. *International Journal of Remote Sensing* **39**, 949–970 (2018).
11. Running, S. & Zhao, M. MOD17A3HGF MODIS/Terra Net Primary Production Gap-Filled Yearly L4 Global 500 m SIN Grid V006 [Data set]. *NASA EOSDIS Land Processes DAAC* (2019) doi:10.5067/MODIS/MOD17A3HGF.006.
12. Atkin, O. K., Meir, P. & Turnbull, M. H. Improving representation of leaf respiration in large-scale predictive climate–vegetation models. *New Phytologist* **202**, 743–748 (2014).

13. Dantas de Paula, M. *et al.* Nutrient cycling drives plant community trait assembly and ecosystem functioning in a tropical mountain biodiversity hotspot. *New Phytologist* **232**, 551–566 (2021).
14. Thum, T. *et al.* A new model of the coupled carbon, nitrogen, and phosphorus cycles in the terrestrial biosphere (QUINCY v1.0; revision 1996). *Geoscientific Model Development* **12**, 4781–4802 (2019).
15. Spawn, S. A. & Gibbs, H. K. Global Aboveground and Belowground Biomass Carbon Density Maps for the Year 2010. *ORNL DAAC* (2020) doi:10.3334/ORNLDAAC/1763.
16. OCHA. *Latin America and the Caribbean: Natural Disasters 2000-2019*. https://www.humanitarianresponse.info/en/operations/latin-america-and-caribbean/document/latin-america-and-caribbean-natural-disasaters-2000 (2020).
17. Lange, S. *et al.* Projecting Exposure to Extreme Climate Impact Events Across Six Event Categories and Three Spatial Scales. *Earth's Future* **8**, e2020EF001616 (2020).
18. World Bank. *GDP (current US$). Dataset ID: NY.GDP.MKTP.CD*. https://data.worldbank.org/indicator/Ny.Gdp.Mktp.Cd (2021).
19. World Bank. *GDP per capita (current US$). Dataset ID: NY.GDP.PCAP.CD*. https://data.worldbank.org/indicator/NY.GDP.PCAP.CD (2021).
